# Multivalent effect of peptide functionalized polymeric nanoparticles towards selective prostate cancer targeting†

**DOI:** 10.1039/d2na00601d

**Published:** 2023-01-17

**Authors:** Madhura Murar, Silvia Pujals, Lorenzo Albertazzi

**Affiliations:** a Institute for Bioengineering of Catalonia (IBEC), The Barcelona Institute of Science and Technology (BIST) Barcelona Spain l.albertazzi@tue.nl silvia.pujals@iqac.csic.es; b Institute for Advanced Chemistry of Catalonia (IQAC) Barcelona Spain; c Department of Biomedical Engineering, Institute of Complex Molecular Systems (ICMS), Eindhoven University of Technology Eindhoven The Netherlands

## Abstract

The concept of selective tumor targeting using nanomedicines has been around for decades; however, no targeted nanoparticle has yet reached the clinic. A key bottleneck is the non-selectivity of targeted nanomedicines *in vivo*, which is attributed to the lack of characterization of their surface properties, especially the ligand number, thereby calling for robust techniques that allow quantifiable outcomes for an optimal design. Multivalent interactions comprise multiple copies of ligands attached to scaffolds, allowing simultaneous binding to receptors, and they play an important role in targeting. As such, ‘multivalent’ nanoparticles facilitate simultaneous interaction of weak surface ligands with multiple target receptors resulting in higher avidity and enhanced cell selectivity. Therefore, the study of weak binding ligands for membrane-exposed biomarkers is crucial for the successful development of targeted nanomedicines. Here we carried out a study of a cell targeting peptide known as WQP having weak binding affinity for prostate specific membrane antigen, a known prostate cancer biomarker. We evaluated the effect of its multivalent targeting using polymeric NPs over its monomeric form on the cellular uptake in different prostate cancer cell lines. We developed a method of specific enzymatic digestion to quantify the number of WQPs on NPs having different surface valencies and observed that increasing valencies resulted in a higher cellular uptake of WQP-NPs over the peptide alone. We also found that WQP-NPs showed higher uptake in PSMA over-expressing cells, attributed to a stronger avidity for selective PSMA targeting. This kind of strategy can be useful for improving the binding affinity of a weak ligand as a means for selective tumor targeting.

## Abstract 2

Multivalent nanoparticles facilitate simultaneous interaction of weak surface ligands with multiple target receptors resulting in higher avidity and enhanced cell selectivity.

## Graphical abstract

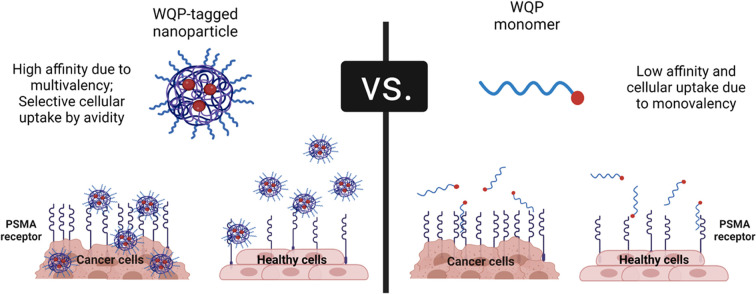

## Introduction

Prostate cancer (PCa) is the most commonly diagnosed malignancy and the second most prevalent cause of cancer deaths in males worldwide.^1^ Although conventional therapies like surgery, radiation, and hormone therapy are relatively efficient for the treatment at early stages, most patients with metastatic PCa ultimately experience a relapse.^2^ Chemotherapy and immunotherapy are currently widely used for advanced prostate cancer treatment, but with limited success, essentially leading to a poor prognosis.^3^ The lack of targeted delivery continues to be a bottleneck that limits the effectiveness of chemotherapy,^4^ and as a result, requires a great deal of attention for the development of efficient targeted drug delivery systems for prostate cancer treatment.^5^

Prostate specific membrane antigen (PSMA) is a 100 kDa type II transmembrane glycosylated protein with folate hydrolase activity and is overexpressed not only in nearly all prostate cancer cells,^6^ but also in tumor neovasculature in a variety of cancers.^6–8^ In contrast, it has minimal expression (100–1000 times lower) in normal prostate epithelium tissues and other normal tissues,^7,9^ making it an ideal biomarker for the design of various targeted therapies. Several different kinds of targeting ligands against PSMA have been discovered over the years;^10–12^ however, they have not yet had a successful clinical translation, owing to multiple factors, namely, high production cost, low shelf life and blood clearance rate, and immunogenicity.^12,13^ Although antibodies and aptamers are the most commonly used ligands having high binding affinity to PSMA,^10^ small cancer cell targeting peptides (CTPs) have a number of advantages, including small molecular weight, high permeability, improved stability, less immunogenicity, ease of synthesis and flexibility in chemical conjugation.^4,14–16^ One such example is the WQP peptide, which is 12 amino acids long, having the sequence WQPDTAHHWATL, and is known to have a moderate/low binding affinity to the extracellular domain of PSMA, which can be further enhanced up to 10-fold, by its dimerization.^17^ Even though multiple studies had previously shown that the peptide-binding affinity can be improved by increasing the binding avidity through use of multivalent binding strategies, such as dimeric or tetrameric peptides or streptavidin–biotinylated peptide tetramers,^17–20^ nanoparticle-mediated multivalent WQP targeting studies still remain scarce.

Nanoparticles (NPs) have emerged as a promising platform in the past two decades encompassing aspects of targeted diagnosis and therapy within the same system, predominantly in the field of cancer treatments.^21,22^ One of the main advantages of the use of NPs for targeted drug delivery is their ability to functionalize surfaces with a wide range of targeting moieties such as antibodies, aptamers, small peptides, *etc.*,^23^ thus providing a tuneable multivalent platform for active targeting.^24,25^ Despite consistent attempts being made for the design of optimal targeted nanomedicines, only a small fraction (∼0.7%) is found to reach the target site.^26^ This drawback can be attributed to the lack of robust characterization strategies for the number and functionality of the attached surface ligands.^27,28^ Most of the common methods employed for the characterization of ligand functionalization depend on bulk results, failing to take into account the inter- and intra-particle heterogeneity in surface ligand number and distribution, which has a direct consequence on its biological response.^29,30^ Therefore, there is a clear need for characterization techniques that allow robust quantification of surface ligands.

Surface properties such as the valency plays an important role in the determination of targeting potential of NPs and their subsequent cellular fate.^31,32^ In the last few years, there has been a great deal of attention on the development of multivalent NPs for their improved biological performance.^27,33^ The multivalency allows for simultaneous binding to multiple receptors, which varies sharply with receptor concentration, thus allowing for selective targeting of tumor cells,^34^ which is the preferred strategy moving towards personalized nanomedicine.^33^ Furthermore, achieving super-selective targeting also revolves around the use of weak-binding ligands,^24^ which allow binding only to a specific density of target receptors, thus providing a way to avoid undesired off-site responses.^35^

Within this framework, we designed a study to test the effect of multivalent targeting of the WQP peptide-functionalized polymeric NPs in comparison to its weak-binding monomeric counterpart on the cellular uptake across different PCa and healthy cell lines. We synthesized and characterized a WQP-Cy5 monomer along with multivalent WQP-NPs having varying surface WQP valencies (5% and 30%). We developed the enzymatic digestion technique, which is routinely used in quantification of proteins, for the purpose of specifically quantifying a surface WQP peptide in a robust manner. We then tested the cellular uptake of all formulations in all cell lines with varied expression levels of PSMA. Our results show that multivalent NPs have higher cellular uptake than the monomer owing to the improved affinity by virtue of simultaneous targeting. Furthermore, we demonstrate that the multivalent NPs show higher selectivity by targeting only those cells with overexpression of PSMA. These studies pave a path for an optimal design of nanosystems for selective targeting of PCa.

## Experimental section

### Materials for WQP-Cy5 peptide synthesis

Unless stated otherwise, all solvents were obtained from commercial sources in at least analytical quality (a.r.) and were used without further purification. Ultrapure water was obtained from a Milli Pore system from Merck. 2-Chlorotrityl chloride resin along with all the Fmoc-l-amino acids were obtained from Iris Biotech. HBTU was purchased from Chempep. DIEA, piperidine, and 2,4,6-trimethylpyridine were obtained from Sigma-Aldrich. An *O*-ninhydrin test kit was purchased from Anaspec. Sulfo-cyanine5 maleimide (sulfo-Cy5 mal) was purchased from Lumiprobe. The reducing agent tris(2-carboxyethyl)phosphine hydrochloride (TCEP) 0.5 M pH 7 was supplied by Merck Life Science.

### Materials for WQP-NP conjugation, formulation, and surface characterization

The polymer poly(lactide-*co*-glycolide)–methoxypoly(ethylene glycol) (*M*_w_ PLGA : PEG, 30 : 1 kDa, L : G in PLGA 50 : 50) was supplied by GenoTech. The polymers poly(lactide-*co*-glycolide) acid endcap (PLGA, 50 : 50 LA : GA, *M*_w_ 25–35 kDa) and poly(lactide-*co*-glycolide)–poly(ethylene glycol) (*M*_w_ PLGA : PEG 30 : 5 kDa, L : G in PLGA 50 : 50) were supplied by PolySciTech. The polymer poly(d,l-lactide-*co*-glycolide)–poly(ethylene glycol)–maleimide (*M*_w_ PLGA : PEG : maleimide 20 : 5 : 0.09707 kDa) was supplied by Nanosoft Biotechnology LLC. Amicon Ultra-4 and Ultra-2 filters (regenerated cellulose, 100 kDa) were purchased from Merck Life Sciences. The Thermo Scientific™ Snakeskin™ dialysis tube 10K MWCO, and Pierce™ chymotrypsin protease (TLCK treated), MS grade were obtained from Fisher Scientific. The solvent acetonitrile (ACN) (HPLC grade) was purchased from Serviquimia.

### Materials for cellular uptake studies

All PCa cell lines were cultured in RPMI-1640 medium with a Glutamax supplement (Life Technologies) and supplemented with 10% fetal bovine serum (FBS) and an antibiotic mixture of 1× penicillin/streptomycin obtained from Life Technologies and LabClinics, respectively. RWPE1 cells were cultured in Keratinocyte serum free medium supplemented with bovine pituitary extract (BPE) and epithelial growth factor (EGF), obtained from Life Technologies. Cells were seeded in Nunclon™ (Nunc) Delta surface treated 6-well plates and 8-well chamber slides (Lab Tek) obtained from Thermosfisher for flow cytometry analysis and confocal imaging, respectively.

## Methods

### Synthesis of the WQP-Cy5 monomer

The WQP peptide was synthesized by solid phase peptide synthesis (SPPS) using the 9-fluorenylmethoxycarbonyl/*tert*butyl (Fmoc/*t*Bu) strategy.^36,37^ 2-Chlorotrityl resin, l-Fmoc-protected amino acids (2 equiv.), coupling agent TBTU (2 equiv.), and DIEA (6 equiv.) were used. The Fmoc protecting group was cleaved by treatment with a solution of 20% piperidine in DMF (2 × 10 min). Peptides were cleaved from the resin by treatment with reagent B (88% TFA, 2% TIPS, 5% water and 5% phenol) for 4 h. The crude peptide obtained was then purified at a wavelength (*λ*) of 280 nm by semipreparative RP-HPLC-MS [Waters 2487 Dual Absorbance Detector equipped with a Waters 2700 Sample Manager, a Waters 600 Controller, a Waters Fraction Collector, a Symmetry column (C18, 5 mm, 30 × 100 mm)] using the MassLynx software. HPLC conditions: flow = 6 mL min^−1^; gradient = 30–60% B in 5 min; A = 0.1% TFA in H_2_O, B = 0.05% TFA in ACN. A total of 40% yield of pure WQP was obtained.

Part of the purified peptide was then conjugated to sulfo-Cy5 maleimide using the maleimide-thiol reaction using the protocol provided by the manufacturer (ThermoFisher Molecular Probes (B7884)). Briefly, 5 mg of pure WQP peptide in 1 mL of 1× PBS (pH 7.4) was first treated with 10× molar excess of TCEP solution for 20 minutes at R.T. under stirring conditions (to reduce thiol groups). Next, 5 mg of sulfo-Cy5-maleimide was dissolved in 500 μL of DMSO to have a final molar ratio of 1 : 2 of thiol : maleimide and added dropwise to the peptide solution. The reaction was allowed to take place for 2 hours at room temperature with constant stirring. Unconjugated dye was removed in the form of a supernatant *via* centrifugation using Amicon Ultra-4 (3 kDa) filters as per filter instructions for 10 min at 5000×*g* (rcf) at 20 °C with filtered MiliQ water. Conjugated WQP-Cy5 was then purified using semi-preparative HPLC-MS (similar conditions as WQP purification), and the obtained sample was stored in MiliQ water in the dark at 4 °C until further use.

### Polymer–peptide pre-conjugation

A previously purified WQP peptide was pre-conjugated to the PLGA_30k_–PEG_5k_–maleimide polymer using an organic solvent (ACN) and incubated under stirring conditions overnight. Next, the conjugate was purified from unreacted polymer by precipitation using an ice-cold diethyl ether/methanol (DEE/MeOH) mixture. The precipitate obtained was washed twice with the DEE/MeOH mixture and finally redissolved in ACN and dialyzed for a minimum of 24 hours against pure ACN to separate the unreacted WQP from the conjugate. Next, the purified conjugate was lyophilized and a final yield of approximately 35% was obtained. Thereafter, 5 mg of polymer, WQP peptide and the conjugate each were dissolved in 0.7 mL of deuterated dimethyl sulfoxide (d_6_-DMSO) and characterized using Bruker 500 Hz proton nuclear magnetic resonance (^1^H NMR) and analyzed using the Mnova (v.14.0) software.

### Multivalent nanoparticle formulation

Multivalent PLGA–PEG–WQP NPs were formulated *via* the nanoprecipitation method according to the literature.^38^ Briefly, 3 mg of polymer mixture and 1.1 mM of 1,1′-dioctadecyl-3,3,3′3′-tetramethylindocarbocyanine perchlorate (DiI) were dissolved in 300 μL solvent phase (ACN) at room temperature. The PLGA polymer was maintained at a ratio of 15% and mixed with PLGA_30k_–PEG_1k_ and PLGA_30k_–PEG_5k_–WQP conjugate at 5% or 30% of surface WQP valencies. For control PLGA–PEG formulations, the PLGA–PEG–WQP-conjugate was substituted with PLGA–PEG–Mal, whilst the PLGA amount was maintained constant at 15%. The anti-solvent phase (MiliQ water) was stirred at 200–300 rpm whilst the solvent phase comprising the polymer solution (ACN) was pipetted at a 1 : 10 ratio (300 μL polymer solution is pipetted into 3 mL MiliQ water). Solvent extraction (evaporation) was continued for 5 h under magnetic stirring in a fume hood at room temperature. NPs were then collected by high-speed centrifugation (Avanti J-26 XPI, rotor JA-14) using Amicon Ultra-4 100 kDa filters as per filter instructions (10 min at 5000×*g* at 20 °C) with filtered MiliQ water. NPs were stored in MiliQ water at a 10 mg mL^−1^ concentration in the dark at 4 °C until further use.

### Surface peptide quantification by enzymatic digestion

The quantification of surface WQP was carried out using chymotrypsin protease by following the protocol provided by the manufacture (Pierce™, MAN0011638) with certain optimizations. Since the protocol is usually used for protein samples, the WQP-NP samples were concentrated up to 25–50 mg mL^−1^ so as to have enough concentration of the digested peptide fragments to be detected by mass spectrometry. The digestion reaction was carried out using an enzyme: peptide sample ratio of 1 : 20, in the digestion buffer [500 mM Tris HCl (pH 8.0) + 10 mM CaCl_2_]. The reaction vessel was incubated at 37 °C overnight and the digested fragments were collected from the supernatant obtained by briefly (5 min) centrifuging the samples to separate the NPs, and they were then characterized by UPLC-MS at *λ* = 280 nm as a peak corresponding to the mass of the chosen digested fragment.

### Qualitative analysis of cellular uptake by confocal laser scanning microscopy (CLSM)

All cell lines (LNCaP, PC3, 22Rv1 and RWPE1) were cultured in an 8-well LabTek (25 × 10^3^ cells per well) for 24 h at 37 °C and 5% CO_2_, until they reached up to 70% confluence and then incubated with a known concentration (50 μg mL^−1^) of the WQP-Cy5 monomer, and 5% and 30% multivalent WQP-NPs dissolved in a culture medium without serum (RPMI 1640 for LNCaP, PC3 and 22Rv1; and KSFM for RWPE1) for 24 h at 37 °C and 5% CO_2_. Post incubation, the medium containing the WQP-monomer and multivalent NPs was aspirated, and the cells were stained with the nuclear dye Hoechst33342 (1 μg mL^−1^) for 10 minutes at room temperature. The cells were then imaged live at 37 °C and 5% CO_2_ using the 63× oil immersion objective of a Zeiss LSM 800 confocal microscope. The cell nuclei, WQP-Cy5 monomer and DiI encapsulated WQP-NPs were excited using 405 nm, 640 nm and 561 nm lasers, respectively.

### Quantitative analysis of cellular uptake by flow cytometry

For flow cytometry, all the cell lines were cultured in 6-well plates (1 × 10^5^ cells per well) for 24 h until they reached up to 70% confluence and then incubated with a known concentration (50 μg mL^−1^) of WQP-Cy5 monomer, and 5% and 30% multivalent WQP-NPs for 24 h at 37 °C and 5% CO_2_. Post incubation with NPs, the adherent cells were detached using 0.25% trypsin/EDTA, incubated for 10 minutes at 37 °C and 5% CO_2_, and obtained in a suspension by centrifugation at 3000 rpm for 5 minutes at 4 °C. From this moment onwards, all the further steps like washing with 1× PBS and resuspension in 1× PBS, were carried out whilst maintaining the cells on ice. Finally, the cells were resuspended in 1× PBS solution with the live cell staining agent DAPI (10 μg mL^−1^) and analysed using BD FACSAria™. The cells not stained with DAPI were excluded from the analysis. At least 10 000 cells (or events) were analysed using two specific lasers for WQP-Cy5 (Red C-670 nm) and multivalent WQP-NPs encapsulated with DiI (Green E-575 nm). All measurements were carried out in triplicate and the standard deviation was obtained.

## Results and discussion

### Mono and multivalent WQP formulation

**Fig. 1:**
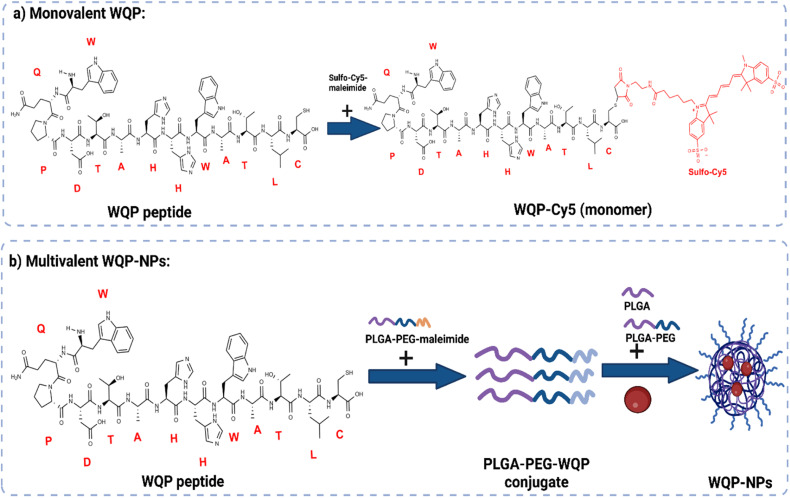
Scheme of mono and multivalent WQP synthesis. (a) WQP peptide is conjugated to sulfo-Cy5-maleimide dye by maleimide–thiol chemistry. (b) WQP peptide is conjugated to the PLGA–PEG–maleimide polymer and PLGA–PEG–WQP NPs are formulated by nanoprecipitation.

The WQP peptide was synthesized using SPPS and modified at the non-PSMA binding C-terminal using a cysteine amino acid having a free thiol group. For the monovalent form of the peptide (Fig. 1a), the synthesized WQP was conjugated with a thiol-reactive fluorescent dye, sulfo-cy5-maleimide, using maleimide–thiol chemistry. The success of conjugation was characterized using ultra-performance liquid chromatography-mass spectrometry (UPLC-MS), followed by purification of the peptide–dye conjugate (Fig. S1†).

For multivalent NP formulations (Fig. 1b), the peptide was first conjugated to the PLGA–PEG–maleimide polymer using maleimide–thiol chemistry in an organic solvent overnight under stirring conditions. This was then followed by formulation of multivalent NPs having different WQP surface valencies (5% or 30%) by the manual process of nanoprecipitation. The formulated NPs were characterized for size and surface charge using transmission electron microscopy (TEM) and dynamic light scattering (DLS).

### Multivalent WQP-NP pre-conjugation, formulation, and characterization

**Fig. 2:**
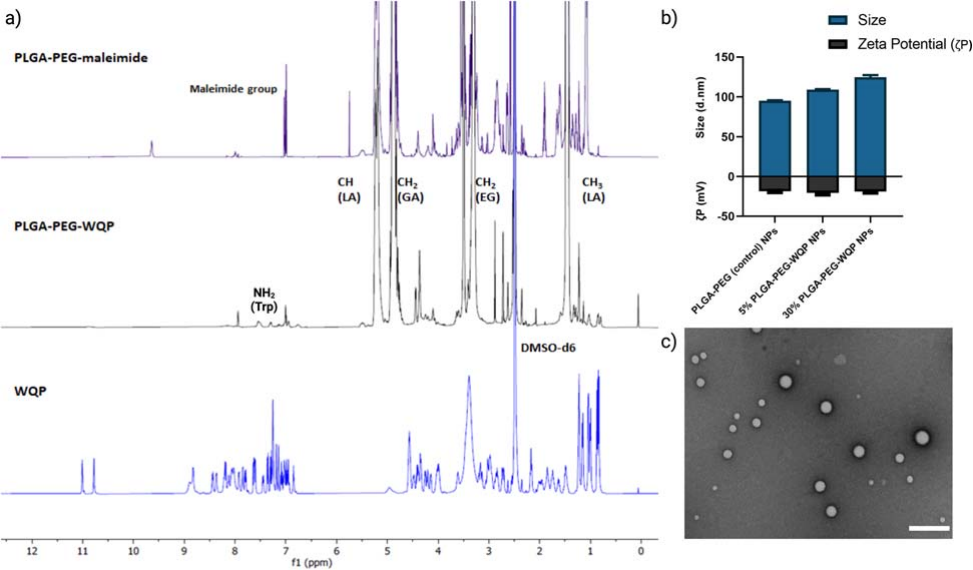
Polymer–peptide conjugation, and NP formulation and characterization. (a) Characterization of PLGA–PEG–WQP conjugation by ^1^H NMR: overlapped spectra with coinciding regions shown. (b) Multivalent WQP-NPs formulated from the WQP conjugate characterized for size (nm) and *ζ*-potential (mV) using DLS and (c) TEM image of 30% WQP-NPs at scale bar 200 nm.

We pre-conjugated the WQP peptide to the PLGA–PEG–maleimide polymer under organic conditions and then purified it using precipitation and dialysis processes to finally obtain the PLGA–PEG–WQP conjugate. Next, we characterized the extent of this conjugation reaction using ^1^H NMR, which allowed for obtaining the spectra for hydrogen nuclei specific for the polymer (PLGA–PEG–Mal), the peptide (WQP), and the conjugate (PLGA–PEG–WQP) as shown overlapped in Fig. 2a. The intense signals observed at *d* = 1.46, 4.9 and 5.2 ppm in the PLGA–PEG–Mal and PLGA–PEG–WQP spectra correspond to the methyl (–CH_3_), methylene (–CH_2_) and methine (–CH) groups of PLGA. On the other hand, the signals observed in the PLGA–PEG–WQP and pure WQP spectra at around *d* = 7.31 and 7.53 ppm are attributed to the protons from the amide group (–NH_2_) present in the amino acid tryptophan (W) of the peptide, thereby confirming its successful conjugation to the polymer. After integrating the area under the peaks corresponding to the protons of the WQP peptide with those from the polymer (Fig. S2†), the conjugation efficiency (CE) was calculated. We found that the pre-conjugation of WQP to the polymer allowed for a CE of 77% (Table S1†), so it was chosen as the preferred strategy.

We then formulated multivalent NPs having varied surface WQP valencies (5% and 30%) using the PLGA–PEG–WQP conjugate along with combinations of PLGA and PLGA–PEG co-polymers manually by the nanoprecipitation process.^39^Fig. 2b and c show the characterization of the formulated multivalent NPs in terms of size and morphology using dynamic light scattering (DLS), transmission electron microscopy (TEM) and net surface charge using zeta (*ζ*) potential. We found a small increasing trend in the sizes of non-targeted PLGA–PEG NPs, 5% and 30% PLGA–PEG–WQP NPs, owing to increasing numbers of WQP on the NP surface (90–120 nm). However, given the small size of the peptide (13 amino acids), the increase in size is rather small (10–20 nm). Similarly, we see a small increase in the surface negative charge of PLGA–PEG NPs by addition of the WQP peptide (18–20 mV) which has a net charge of (−0.9) at physiological pH (7.0). In terms of NP morphology by TEM, we obtained spherical multivalent WQP-NPs with an average diameter of 95 nm. The decrease in the diameter obtained by TEM can be attributed to the fact that DLS measures the hydrodynamic radius, while TEM measures naked particles (under dry conditions).

### Quantification of WQP on the NP surface by specific enzymatic degradation

As explained before, one of the main reasons behind inefficiency of targeted nanomedicines is the lack of robust characterization of surface ligands. Routinely employed techniques include some spectrometric or colorimetric tests,^40^ but these have limited utility in terms of the type of ligand used, in that, most of them are known to work predominantly for measuring larger molecules like proteins, antibodies, *etc.*^41^ Furthermore, given the strengths and weaknesses of each technique, in most cases, a combinatorial approach has to be employed,^40^ which is time-consuming and not always efficient. In this context, we introduce the use of specific enzymatic digestion to quantify the number of surface peptides in a robust manner. Again, this method is routinely used for protein quantification,^42^ but we exploit its high sensitivity and show that it can also be easily used for quantification of small peptides.

**Fig. 3:**
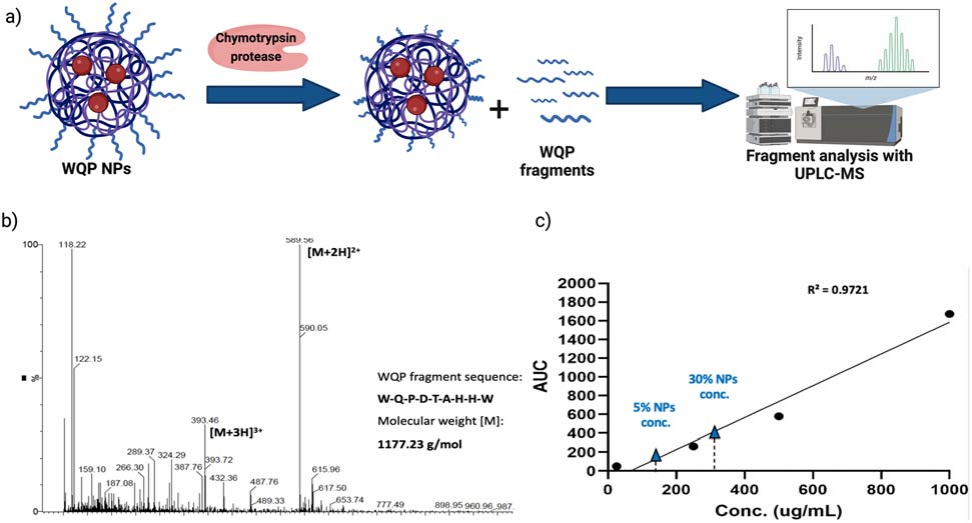
Enzymatic degradation of WQP-NPs and quantification of surface WQP. (a) Chymotrypsin protease specifically cleaves at the C-terminal of aromatic amino acids (tryptophan-W), resulting in digested fragments of the surface WQP peptide, one of which is then chosen for analysis using UPLC-MS. (b) UPLC-MS spectrum of the chosen digested WQP fragment and (c) calibration curve of the digested WQP fragment with varying concentrations used for calculation of the number of WQP on the NP surface.

Proteases are a type of enzyme that specifically cleave amino acid sequences in proteins.^43^ Chymotrypsin protease is such an example, and it is known to specifically cleave at the C-terminal of aromatic amino acids such as tryptophan (W), tyrosine (Y) and phenylalanine (F).^44^ In the sequence of the WQP peptide (WQPDTAHHWATLC), we expected a highly specific cleavage at the C-terminal end of tryptophan (W) at position 9 (starting from N-terminal). We confirmed the presence of the digested fragment having a molecular weight of 1177.23 Da by analysing the supernatant obtained after pelleting down the NPs and using UPLC-MS as shown in the schematic in Fig. 3a. The high sensitivity of UPLC-MS allowed us to detect the digested fragment as a distinct peak, which we integrated to obtain the corresponding area on the chromatogram at *λ* = 280 nm (Fig. S3†), which showed masses specific to the fragment divided by charge of the protons (+2 and +3), in the corresponding spectra shown in Fig. 3b. We then prepared a calibration curve using a range of concentrations of the digested peptide alone to calculate the number of WQP from the concentration (μg mL^−1^) of unknown samples as shown in Fig. 3c. As demonstrated in Table S2,† expectedly, 5% NPs had a lower number of WQPs on the surface in comparison to 30% NPs, owing to an increase in surface valency. However, somewhat counterintuitively, 5% NPs showed a better surface coverage, in that, 91% of WQP was found on the NP surface in comparison to 53% of WQP on the surface of 30% NPs. These changes in surface coverage of WQP could be attributed to the steric hindrance caused by overcrowding of the surface with ligands in NPs with higher ligand density (30%), which could possibly result in embedding of WQP in the NP core, subsequently causing a decreased number of WQP peptide on the NP surface for reaction with the enzyme.

Thus, we see that the expected surface WQP number and its coverage are not linearly correlated. And in order to determine the ideal surface valency to observe a multivalent effect, both these properties need to be accounted for, pushing forth the parameters for rational design of effective targeted NPs.

### Effect of mono *versus* multivalent WQP-mediated uptake in PCa cell lines by CLSM and flow cytometry

**Fig. 4:**
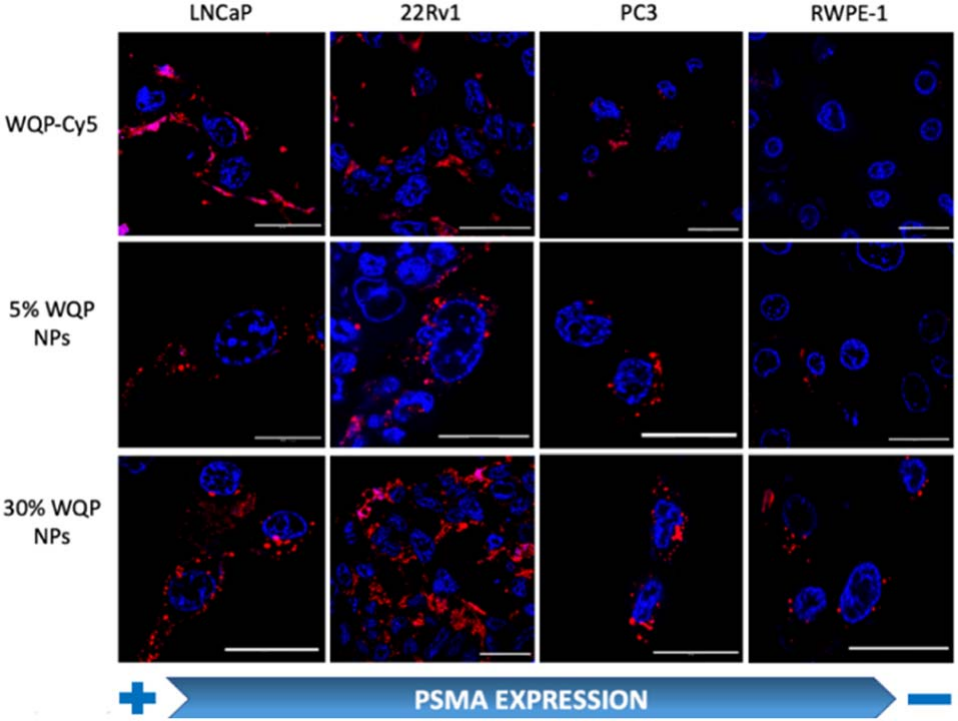
Multivalency effect of WQP-NPs on cellular uptake by confocal imaging. Cellular uptake of the WQP-monomer (tagged with Cy5-red) and multivalent WQP-NPs (encapsulated with DiI-red) having different surface WQP densities (5% and 30%) across different prostate (healthy and cancerous) cell lines post 24 h of incubation analysed by confocal laser scanning microscopy. Cellular nuclei tagged with Hoechst33342 (blue). The scale bar is 25 μm.

For testing the multivalent effect of the WQP peptide on cellular uptake, we used four prostate (cancer-LNCaP, PC3 and 22Rv1; healthy-RWPE1) cell lines having varying levels of PSMA expression (Fig. S4a†). The cells were incubated with 50 μg mL^−1^ of both the WQP-Cy5 monomer and (5% and 30%) WQP-NPs in serum-free media for 24 h at 37 °C, 5% CO_2_. The use of serum-free media was to ensure that the effect on uptake of WQP-tagged NPs was a result of multivalency alone, and not biased due to protein corona formation which is usually observed in the case of serum containing media. Next, we imaged all the cell lines using CLSM for qualitatively analysing the cellular uptake of WQP-tagged fluorophores (Cy5 from the monomer and DiI encapsulated within the NPs) (Fig. 4).

As expected, we observed an increased cellular uptake in the case of multivalent WQP-NPs, with higher uptake by NPs with higher surface WQP valency (30% WQP-NPs), in comparison to the WQP-Cy5 monomer. This increase in uptake can be attributed to the multivalent targeting by WQP-tagged NPs. Furthermore, we observed a higher uptake in cells with higher PSMA expression (LNCaP and 22Rv1) over those with lower PSMA expression (PC3 and RWPE1), thereby allowing for selective targeting of PCa cells, a desirable property in nanomedicine targeting strategies (Fig. 4).

Flow cytometry is a known robust technique that works on the principle of light scattering and fluorescence emission by the specific fluorescent probe-labelled cells as they pass through a laser beam. It offers several unique advantages as it allows fast, relatively quantitative, multiparametric analysis of cell populations at the single cell level.^45^ We used flow cytometry to quantify and compare the uptake of multivalent-WQP NPs over that of the peptide alone across different PCa cell lines.

**Fig. 5:**
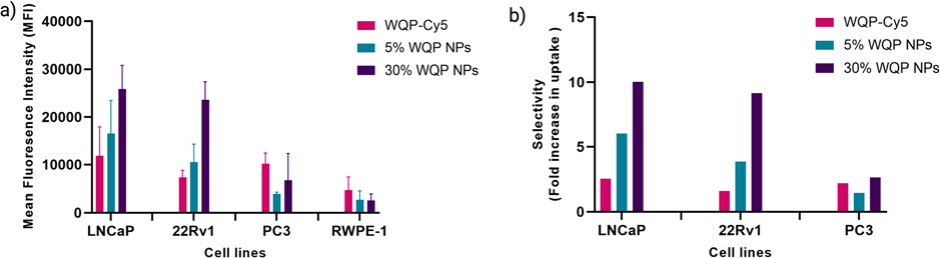
WQP-NP mediated selective cellular uptake. (a) Cellular uptake of monomeric WQP-Cy5 and multivalent WQP-NPs (5% and 30%) quantified by flow cytometry. (b) Selective cellular uptake (fold increase) of multivalent WQP-NPs and WQP-Cy5 monomer in comparison to healthy prostate cells in PCa cell lines.

Fig. 5a shows a clear overall increase in cellular uptake of multivalent WQP-NPs with varying surface valencies in comparison to the WQP monomer in LNCaP and 22Rv1 cells having high and moderate PSMA expression. The experiment was performed in triplicate and the standard deviation is plotted. As expected, an increasing trend with regard to increasing surface valencies is observed. This increase in uptake can be attributed to the avidity or the combined strength of the higher number of WQP peptide towards PSMA over-expressing cells. Additionally, the uptake is higher for multivalent NPs with higher surface valency (30% over 5%), thus highlighting the importance of a robust method for quantification of surface WQPs. While there is a possibility of increase in uptake of WQP-tagged NPs due to a passive effect of targeted peptides on the NP surface,^46^ we did not observe it especially in the PC3 and RWPE-1 cell lines, that have lower expression of PSMA. Fig. 5b shows the selective uptake of multivalent WQP-NPs (having 5% and 30% surface valencies) and the WQP monomer in comparison to the healthy RWPE1 cells. What is interesting to note is that the uptake was highly selective in the case of LNCaP and 22Rv1 cell lines, which have higher expression levels of PSMA (Fig. S4†), in comparison to the healthy RWPE-1 cells: 6 and 10-fold increase (LNCaP cells) and 4 and 9-fold increase (22Rv1 cells) from 5% and 30% WQP-NPs respectively, over a 2.5 and 1.5-fold increase from the WQP-Cy5 monomer, thus conferring selectivity through combined strength of the ligand (avidity). This is an important concept in personalization of targeted therapies, with the goal of reducing undesirable effects off-site.

## Conclusions

We formulated stable and monodisperse multivalent WQP-tagged NPs with varying surface valencies and compared their interaction with cells (uptake) to that of the monomeric peptide. We characterized conjugated NPs for surface peptide number using enzymatic digestion and found that while the formulations with lower WQP density have lower number of surface WQP than the higher density formulations, they tend to have a higher surface coverage, a property that must be considered for development of more effective active targeting nano-formulations. Having a lower affinity to the target receptor may be disadvantageous for the monomer, but we successfully demonstrate that this affinity can be improved by multivalent targeting using polymeric NPs. Furthermore, we show that this multivalency allows for selective targeting, a property that is desirable and extremely sought after for the development of more efficient nanomedicines. We believe that these studies help to improve our understanding and would lead to the development of more complex, but effective multi-ligand targeting strategies, paving the way towards personalization of treatments.

## Author contributions

The manuscript was written through contributions of all authors.

## Conflicts of interest

The authors declare no conflicts of interest.

## Supplementary Material

NA-005-D2NA00601D-s001
