# Supplementary material for: Multivalent effect of peptide functionalized polymeric nanoparticles towards selective prostate cancer targeting
Source: Nanoscale Adv. 2023 Jan 17;5(5):1378–85. doi: 10.1039/d2na00601d (PMC9972852; doi:10.1039/d2na00601d)
Supplement: NA-005-D2NA00601D-s001 [file NA-005-D2NA00601D-s001.pdf]

Electronic Supplementary Material (ESI) for Nanoscale Advances.
This journal is © The Royal Society of Chemistry 2023

Multivalent effect of peptide functionalized polymeric nanoparticles towards selective prostate cancer targeting

Madhura Murar,*[a] Silvia Pujals [b] and Author Lorenzo Albertazzi [a, c]

[a.] Institute for Bioengineering of Catalonia (IBEC), the Barcelona Institute of Science and Technology (BIST), Barcelona, Spain.
[b.] Institute for Advanced Chemistry of Catalonia (IQAC), Barcelona, Spain.
[c.] Department of Biomedical Engineering, Institute of Complex Molecular Systems (ICMS), Eindhoven University of Technology, Eindhoven, The Netherlands.
E-mail of corresponding authors: l.albertazzi@tue.nl; silvia.pujals@iqac.csic.es

- **Supplementary figures and tables:**

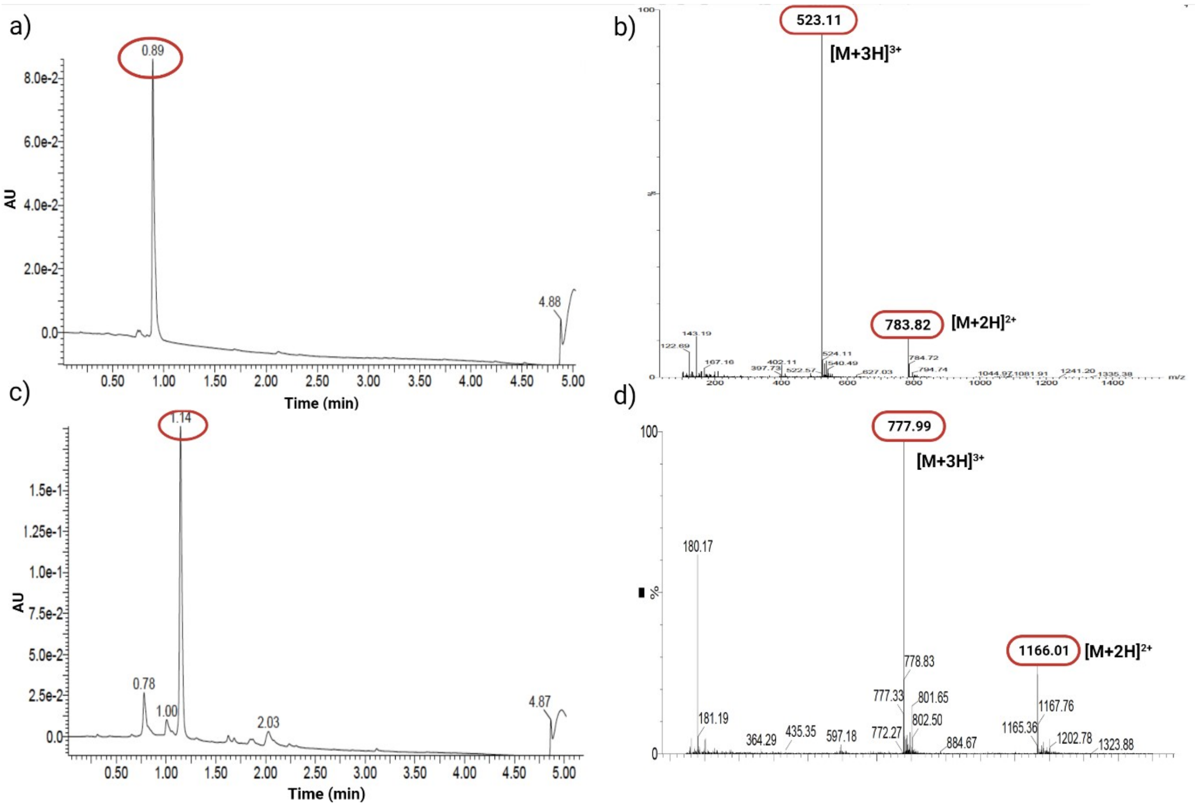

Figure S1. Synthesis and purification of WQP and WQP-Cy5. a) UPLC-MS chromatogram at λ=280nm of purified WQP. b) Mass spectra of purified WQP showing corresponding m/z values. c) UPLC-MS chromatogram at λ=280nm of purified WQP-Cy5 and d) mass spectra of purified WQP-Cy5 showing corresponding m/z values.

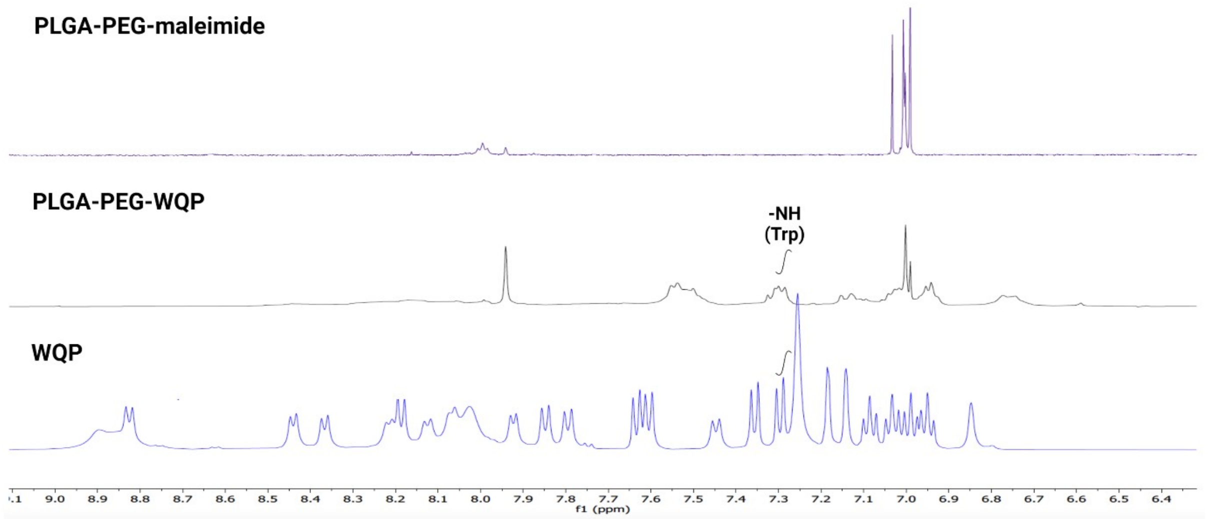

Figure S2. Characterization of peptide-polymer conjugation by ^{1}H NMR. Overlapped zoom-in spectra of the aromatic region (6.5 to 9ppm) of PLGA-PEG-maleimide polymer, WQP peptide and the PLGA-PEG-WQP conjugate. Integrated areas of coinciding peaks (7.31ppm) corresponding to protons from -NH group of the indole ring of tryptophan (Trp) are used for calculation of conjugation efficiency (CE).

Peak	ppm	Integrated area (a_i)	Number of protons (m_i)	Number of repeating units (n_i)	WQP/ PLGA (CE) (%)
-NH (W)	7,31	1,01	1	1	77,1
CH_3 (PLGA)	1,46	300	3	333,33	

Table S1. Calculation of CE by ^{1}H NMR. The conjugation degree of PLGA-PEG copolymer with WQP was calculated by the ratio between the integrated area of the doublet observed at d = 7.31 corresponding to amide protons of tryptophan in the peptide sequence, and that of the integrated peak observed at d = 1.5 (Figure 2), corresponding to the protons of the methyl groups (–CH3) of PLGA.

The CE is calculated as a ratio between WQP/PLGA obtained using the following equation[1]

$$\frac{WQP}{PLGA} = \frac{a_{WQP} / m_{WQP}}{(a_{WQP} / m_{WQP}) + (a_{CH3} / (m_{CH3} \times n_{CH3}))} = \frac{1{,}01/1}{(1{,}01/1) + (300/ (3 \times 333{,}33))} = 0{,}77 \quad (1)$$

where a_i corresponds to the integrated area under the signals of the 1H-NMR spectrum for the respective fractions, m_i corresponds to the number of protons corresponding to each signal, and n_i is the number of repetition units of the fraction i. The calculation indicates that for every PLGA there is 0,77 units of WQP. Thus, the value of CE is 77%.

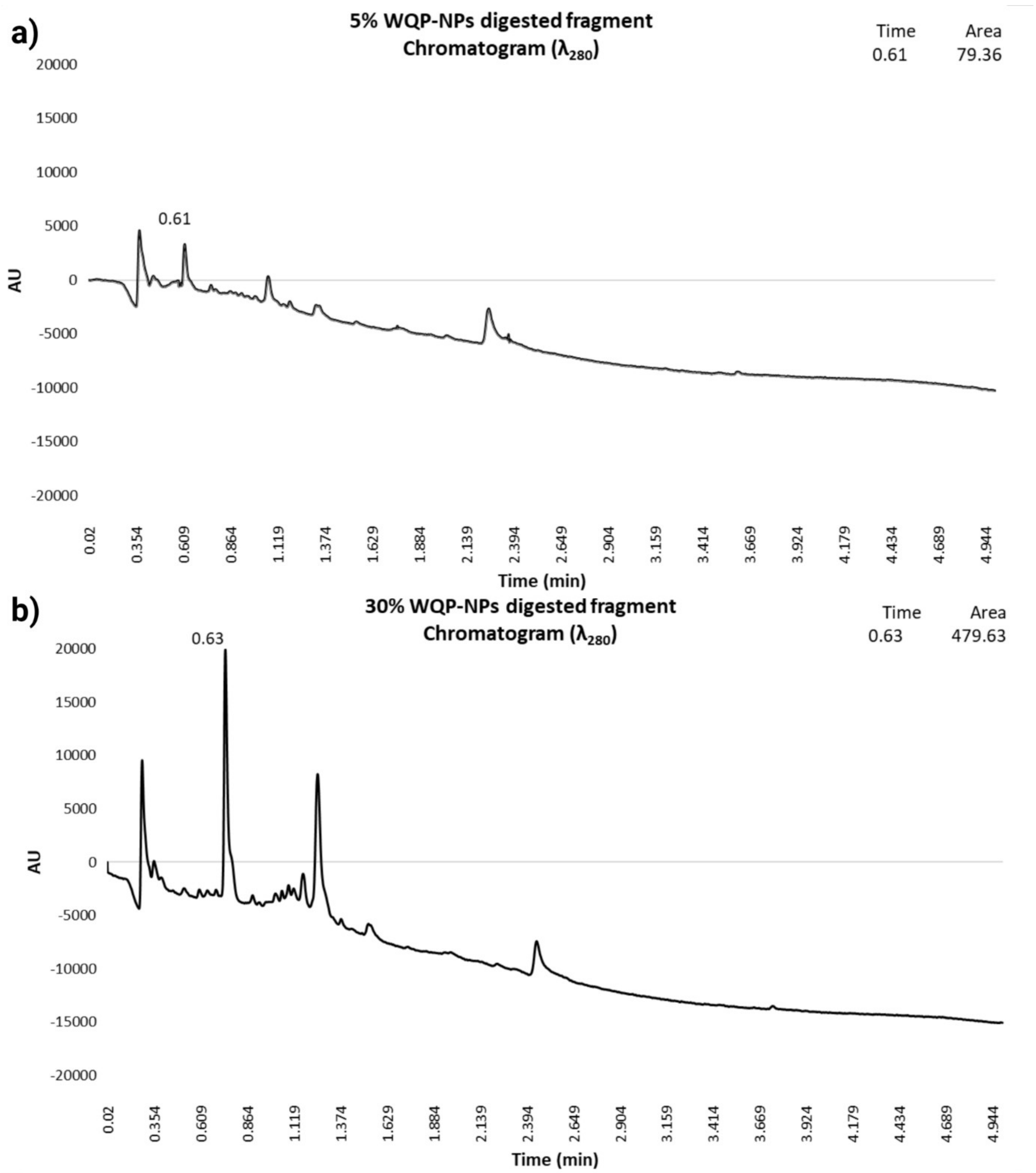

Figure S3. UPLC-MS analysis of surface WQP on multivalent NPs. Integrated chromatograms of digested fragments of multivalent WQP-NPs having different surface valencies a) 5% and b) 30% obtained at λ=280nm.

Calculation of number of WQP on NP surface by enzymatic digestion:

As shown in Figure 3, the digestion of WQP-NPs having different WQP surface densities showed masses corresponding to the digested WQP fragment in the mass spectrum. This peak was integrated, and the area was used to calculate the concentration of WQP in 5% and 30% formulations, using the calibration curve.

For calculation of theoretical (expected) number of WQPs, we used the formula as per Spherotech´s instructions[2] as follows:

1. Calculating the number of NPs in suspension:

   $$\frac{6 \times \text{Polymer weight (g)}}{3.14 \times \text{Polymer density (g/cm3)} \times \text{NP diameter (um)}^3} \times 10^{12} \quad (2)$$

2. Calculating the number of WQP molecules in suspension:

   $$\left(\frac{\text{Mass of WQP-conjugate (g)}}{\text{Molecular weight of WQP-conjugate (g/mol)}}\right) \times \text{Avogadro´s number} \quad (3)$$

3. Calculating the theoretical number of WQP molecules per NP:

   $$\frac{\text{Number of WQP molecules}}{\text{Number of NPs in suspension}} \quad (4)$$

4. Calculation of theoretical WQP per NP: Calculated by multiplying the theoretical WQP molecules/NP *(4)* by the CE % of the formulation *(1)*.
5. Calculation of number of WQP (observed) on NP surface from calibration curve:
   For calculating the number of surface WQP from the concentration (ug/mL) obtained from calibration curve, we plot the integrated area of unknown samples (5% and 30% WQP NPs peaks).
6. Calculating moles of WQP-conjugate added:

   $$\frac{\text{Mass of WQP-conjugate added (g)}}{\text{Molecular weight of WQP-conjugate (g/mol)}} \quad (5)$$

7. Calculation of mass (μg/mL) of WQP added:
   moles of WQP-conjugate added X molecular weight of WQP *(6)*
8. Calculation of moles of WQP obtained on NP surface:

   $$\frac{\text{Mass of WQP (observed)}}{\text{Molecular weight of WQP}} \quad (7)$$

9. Calculation of molecules of WQP on NP surface:
   Moles of WQP (observed) X Avogadro´s number. *(8)*
   We divide this value by molecules of NPs in suspension obtained in (2) to get the number of WQP/ NP.

Samples	Expected #WQP	Observed #WQP	Surface WQP coverage (%)	Actual WQP-valency (%)
5% WQP-NPs	286,07	259	91	4,53
30% WQP-NPs	1716,44	908	53	15,9

Table S2. Calculation of number (#) of WQP on NP surface. The availability (valency and coverage) (%) of WQP on NP surface is calculated by dividing the number of expected versus observed WQP and getting a percentage of the formulated surface valency.

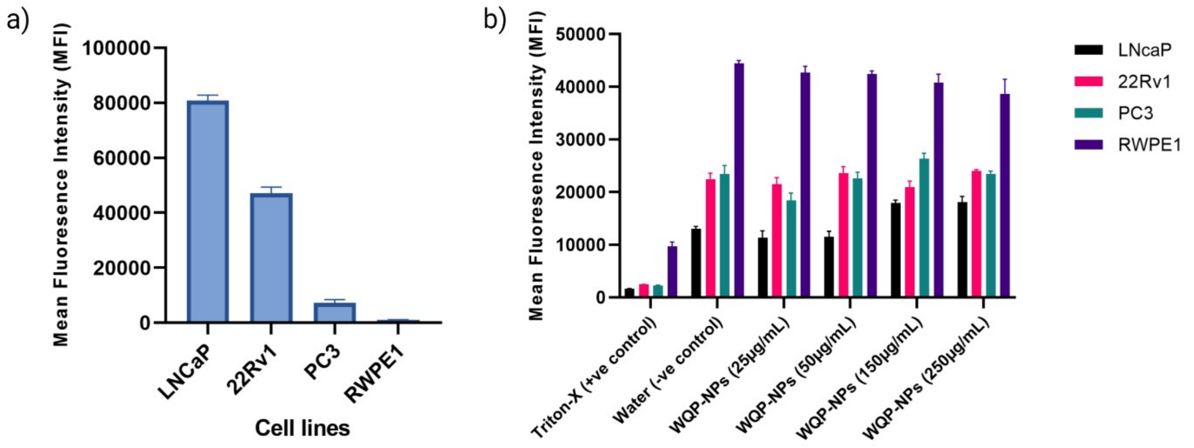

Figure S4. PSMA expression and cellular cytotoxicity of multivalent WQP-NPs. a) PSMA expression across prostate cancer and healthy cell lines obtained by immunostaining as a mean fluorescence intensity of Alexa-488 tagged anti-PSMA antibody using flow cytometry. b) Cellular cytotoxicity of varying concentration ranges of multivalent 30% WQP-NPs on all cell lines by prestoBlue cell viability assay.

For measuring the expression levels of PSMA receptor, all the cell lines were stained with rabbit monoclonal anti-PSMA antibody (ab133579- Abcam Netherlands B.V.) according to manufacturer´s protocol[3]. Briefly 1µg/mL of 1º anti-PSMA antibody dissolved in 3% BSA was added to a monolayer of cells in LabTek on ice for 1hr. Next, the cells were washed thrice with 1X PBS and incubated with 1µg/mL (dilutions provided by manufacturer) of polyclonal goat: anti-rabbit Alexa-488 secondary (2º) antibody for at least 1 hour in dark conditions. For flow cytometry, the cells were detached using 0.25% Trypsin-EDTA incubation for 10 mins at 37ºC, 5% CO_2 and obtained in suspension in 1X PBS. They were then stained with 10ug/mL of DAPI just before analysis with FACS Aria, with the 488nm laser. In total 10,000 cells (or events) were measured, and their mean fluorescence intensity values obtained. Expectedly, LNCaP cells showed highest expression of PSMA followed by 22Rv1 cells having moderate expression and PC3 and RWPE1 cells having low expression.

For measuring the cytotoxicity of the formulated NPs, PrestoBlue cell viability assay was employed as per manufacturer´s instructions[4,5]. Briefly, all cell lines were seeded in a 96-well plate and incubated for 24h at 37ºC and 5% CO_2 . They were then incubated with the formulated multivalent 30%WQP-NPs in varying concentrations (from 25 to 250µg/mL) for 24h at 37ºC and 5% CO_2. As a negative control, filtered MilliQ water of equal volume was used instead of culture medium. Next, the NP-containing medium was aspirated, and the cells were incubated with PrestoBlue (10%v/v of 5% PrestoBlue stock) and incubate for 1hr at 37ºC. Fluorescence was measured at 590nm. Higher fluorescence values were obtained for increasing concentrations of WQP-NPs, which correlated to increase n metabolic activity of the cells, thereby rendering minimal (if none) cytotoxicity for the entire range of concentrations.

References

(1) Hoyos-Ceballos, G. P.; Ruozi, B.; Ottonelli, I.; Da Ros, F.; Vandelli, M. A.; Forni, F.; Daini, E.; Vilella, A.; Zoli, M.; Tosi, G.; Duskey, J. T.; López-Osorio, B. L. PLGA-PEG-ANG-2 Nanoparticles for Blood–Brain Barrier Crossing: Proof-of-Concept Study. *Pharmaceutics* **2020**, *12* (1), 72. https://doi.org/10.3390/pharmaceutics12010072.

(2) *Technical Page - Characteristics of Polystyrene Particles - Spherotech*. https://www.spherotech.com/particle.html (accessed 2022-02-22).

(3) *Indirect flow cytometry (FACS) protocol | Abcam*. https://www.abcam.com/protocols/indirect-flow-cytometry-protocol (accessed 2022-02-22).

(4) Lall, N.; Henley-Smith, C. J.; De Canha, M. N.; Oosthuizen, C. B.; Berrington, D. Viability Reagent, PrestoBlue, in Comparison with Other Available Reagents, Utilized in Cytotoxicity and Antimicrobial Assays. *Int J Microbiol* **2013**, *2013*, 420601. https://doi.org/10.1155/2013/420601.

(5) *PrestoBlue Assays for Cell Viability - ES*. https://www.thermofisher.com/es/es/home/life-science/cell-analysis/fluorescence-microplate-assays/microplate-assays-cell-viability/prestoblue-cell-viability-reagent.html (accessed 2022-07-27).
